# Whole genome sequencing of breast cancer

**DOI:** 10.1111/apm.12920

**Published:** 2019-01-28

**Authors:** Maria Rossing, Claus Storgaard Sørensen, Bent Ejlertsen, Finn Cilius Nielsen

**Affiliations:** ^1^ Centre for Genomic Medicine Rigshospitalet Copenhagen University Hospital Copenhagen Denmark; ^2^ Biotech Research and Innovation Centre University of Copenhagen Copenhagen Denmark; ^3^ Danish Breast Cancer Cooperative Group & Department of Clinical Oncology Rigshospitalet Copenhagen University Hospital Copenhagen Denmark

**Keywords:** Breast cancer, whole genome sequencing, genome maintenance

## Abstract

Breast cancer was the first to take advantage of targeted therapy using endocrine therapy, and for up to 20% of all breast cancer patients a further significant improvement has been obtained by HER2‐targeted therapy. Greater insight in precision medicine is to some extent driven by technical and computational progress, with the first wave of a true technical advancement being the application of transcriptomic analysis. Molecular subtyping further improved our understanding of breast cancer biology and has through a new tumor classification enabled allocation of personalized treatment regimens. The next wave in technical progression must be next‐generation‐sequencing which is currently providing new and exciting results. Large‐scale sequencing data unravel novel somatic and potential targetable mutations as well as allowing the identification of new candidate genes predisposing for familial breast cancer. So far, around 15% of all breast cancer patients are genetically predisposed with most genes being factors in pathways implicated in genome maintenance. This review focuses on whole‐genome sequencing and the new possibilities that this technique, together with other high‐throughput analytic approaches, provides for a more individualized treatment course of breast cancer patients.

Cancer is a disease of the genome and enormous efforts are directed towards understanding of this heterogeneous collection of diseases 1. The expansion of our insight in the cancer genomes is mostly driven by the rapid development in sequencing technologies all the way from the early identification of oncogenes and tumor suppressors to the full annotation of the most common cancers resulting in the so called genomic landscape of cancer 2, 3. Determining the genomic landscape of cancers is an ongoing process supported by The Cancer Genome Atlas (TCGA) dataset which comprises genomic data from >10 000 thousands of tumor samples, providing researchers with a comprehensive catalog of the key genomic changes in more than 30 types of cancer 4. The major advances in sequencing technologies followed by the development of computational tools have enabled analyses like whole‐exome sequencing (WES), RNA‐sequencing (RNA‐seq) and whole‐genome sequencing (WGS) to be implemented in the routine clinical setting, hereby supporting the emerging clinical relevance of genomics in cancer medicine as well as for other diseases 5. The cancer genome is somewhat dynamic, and each cancer evolves with the accumulation of several types of somatic mutations, copy number alterations, epigenetic factors, and structural variants. These changes can occur in a predisposed genetic background like the hereditary cancers which again cause diverse patterns for the individual tumor genome.

Thus, accepting the fact that cancer is a genomic disease and combining this with the growing insights in targeted therapies, the way for precision oncology is being founded. Precision oncology is based on the theory that the examination of both the patients’ genome and the tumor genome will direct the clinician to the targeted drug, expected to be effectual 6. With the implementation of several FDA‐approved HER2‐targted therapies, breast cancer was one of the earliest cancers where implementing targeted therapies have shown a prolonged survival in patients whose tumors are driven by this tyrosine kinase activation 7. Thus, more than 15% of breast cancer patients follow a scheme of targeted therapy, though there is still room for testing and implementation of precision oncology for these patients. In line with this, a recent comprehensive whole‐genome‐based study of both the primary, the locally relapsed, and the metastatic breast cancer showed that cell clones seeding metastasis or relapse disseminate late from the primary tumors, but continue to acquire mutations, mainly within the same pathway as the primary tumor 8. However, Yates et al., found that most distant metastasis acquired new driver mutations, including clinically actionable alterations and mutations, which is highly relevant for the implementation of genome‐driven oncology with the aim of improving the dismal survival of metastatic breast cancer patients.

## Breast cancer

Breast cancer is the most common malignancy among females, and together with lung and colorectal cancers, among the three most common cancers worldwide 9. In 2012 approximately 1.7 million women were diagnosed with breast cancer worldwide, and breast cancer accounts for nearly a third (29%) of all new cancers in women. In women under the age of 50, breast cancer is the leading cause of cancer death, but in elder women it is surpassed by lung cancer as the most frequent cause of death. Overall mortality rates are decreasing although the rates vary greatly worldwide and are the leading cause of cancer death in the less developed parts of the world. The incidence of breast cancer in Denmark is around 4700 new cases per year, and all though mortality rate has decreased mostly attributable to early detection via screening programs and advanced and efficient therapies, breast cancer still causes 1100 deaths per year; being the second cause of cancer death also in Danish women 10.

The diagnostic procedures of breast cancer include clinical examination, breast imaging usually comprising mammography and ultrasound, core‐needle tumor biopsy for histopathology with biomarker assessment, and assignment of intrinsic subtype by molecular genetic analysis (see separate paragraph on molecular subtypes; 11). Based on a clinical risk assessment, presence of comorbidities and patient preferences, a treatment recommendation is for most patients reached by the multidisciplinary team using the biomarker tumor profile of each patient. Algorithms in combination with risk factors have been developed for clinical risk assessment of recurrence and have for Danish breast cancer patients been extended to allow calculation of a prognostic standard mortality rate (SMR) index (PSI) algorithm based on a comprehensive study of >6500 postmenopausal patients with ER positive high‐risk breast cancer 12, 13. Early breast cancer without distant metastasis (Stage I + II) is a possible curative disease with breast conserving surgery in combination with systematic therapy being the standard of care 14. In brief, luminal‐like, ER+ ‐patients are treated with either endocrine therapy alone, or in combination with chemotherapy. If tumors are HER2+, trastuzumab is added. For patients with triple‐negative tumors, standard systemic chemotherapy consists of anthracyclines and taxanes 15.

Neoadjuvant chemotherapy (NACT) – e.g. chemotherapy initiated before breast cancer surgery – is widely used as a standard of care to reduce surgical morbidity of the breast and axilla 16, 17. A recent meta‐analysis substantiated that NACT results in higher rates of breast‐conserving therapy without comprising the risk distant recurrence, breast cancer survival, or overall survival 18. However, uncertainty remains regarding the extend of axillary lymph node assessment and target of radiotherapy after NACT. Targeted therapy directed by tumor profile is a cornerstone in the NACT – setting where a complete pathological response and long‐term outcome is superior to the HER2‐positive or the ER‐negative and HER2‐normal tumors in comparison to the luminal breast cancers for which the response to NACT is more unclear 19.

## Molecular subtypes

Transcriptome analysis enables classification of breast tumors into intrinsic molecular subtypes which are biologically distinct entities with specific prognostic and therapeutic features 20, 21, 22, 23, 24, 25. The pivotal studies proposed five subclasses: (i) the ER‐receptor positive and human epidermal growth factor receptor 2 (HER2)‐receptor negative tumors i.e., luminal A (lumA), luminal B (lumB) and normal breast‐like subclass, (ii) the HER2‐receptor positive tumors: HER2‐like subclass, and (iii) the ER‐ and HER2‐receptor negative tumors called the basal‐like (basL) subclass. Four of the subclasses can be distinguished by a 50‐gene molecular classifier (PAM50) which has been developed as a commercial FDA approved platform (ProsignaVR; 26). This was followed by the 70‐gene signature; *MammaPrint*, which together with a clinical risk evaluation, showed promising results for predicting the low‐risk cancers that may omit chemotherapy 27. In line with this, a comprehensive, multicenter analysis, TAILORx, freshly showed that the 21‐gene recurrence‐score assay (Oncotype DX, Genomic Health) providing prognostic information in hormone‐receptor–positive breast cancer in combination with the 21‐gene‐signature, can direct patients with either a very low score to omit chemotherapy or patients with a high score to enroll for chemotherapy 28. However, for most patients (71%) who were assigned with a mid‐range recurrence score, the TAILORx‐study could not clarify whether disease‐free survival or overall survival was correlated was patients who underwent treatment with chemotherapy or not; this based on 9 years of follow‐up. Recent taxonomies optimized the subclasses by applying integrative genomic analysis and Guedj et al. refined the subclasses by introducing six stable molecular subtypes based on genomic rearrangement and the expression of 256 transcripts 29, 30. However, a comprehensive genomic study integrating both genetic and epigenetic alterations concluded that breast cancers, in addition to the intrinsic subtypes and clinical heterogeneity, can be explained by structural variants defining subclusters within the subtypes 31. Subsequent studies into defining subclusters of subtypes has resulted in several new signatures for identifying specific somatic and pathway‐based subclusters among the intrinsic subtypes 32, 33, 34, 35, 36. For example, Lehmann et al., identified six subclusters among the triple‐negative breast cancers; two basal‐like (BL1 and BL2), an immunomodulatory (IM), a mesenchymal (M), a mesenchymal stem‐like (MSL), and a luminal androgen receptor (LAR) subtype. In addition, 17 pathway‐driven subclusters were suggested by Gatza et al., hereby linking the heterogeneity to tentative therapeutic strategies. In many institutions genomic analysis has in recent years become a part of the standard of care for most breast cancer patients. It is well‐established that the biological hallmark of luminal A subtype is low proliferation, high expression of the *ESR1* gene and a favorable clinical outcome 37. Since 2011, the St Gallen international expert consensus panel has recommended merely endocrine therapy in patients with luminal A disease 15, 38. Attempts have been made indirectly to approximate luminal A – like subtype using IHC biomarkers – ER‐ and PGR‐positive, HER2‐negative and low Ki67 protein staining 39, 40. However, classification with only four biomarkers does not entirely recapitulate the intrinsic subtype of breast cancer 37. From 2017, DBCG recommended molecular subtyping for all patients at intermediate risk, and a recent study showed the benefits of applying up‐front routine subtyping of all early breast cancers, hereby identifying both high and low risk patients 41.

## From Sanger to whole‐genome sequencing

Sequencing of nucleic acids is a method for determining the exact order of each nucleotide (C, G, T and A for DNA sequence and C, G, U and A for RNA sequence). The order of these nucleotides in a polynucleotide string is what finally gives the hereditary and biological codes for all human life. Human DNA consists of about 3 billion nucleotides, and more than 99% of those are identical in all people. Thus, science, including medical human research, is conditioned by the ability to determine the order of nucleotides by sequencing. Or as the double Nobel laureate Frederick Sanger has said it once in a more modest quote: ‘…knowledge of sequences could contribute much to our understanding of living matter.’ 42. In 1953 Watson and Crick determined the three‐dimensional structure of the DNA double helix which revolutionized understanding of science 43. However, the ability to decode the order of nucleotides or sequence were time‐consuming and labor‐intensive until 1977, where the establishment of the Sanger sequencing method took place; Selective incorporation of chain‐terminating dideoxynucleotides by DNA polymerase during *in vitro* DNA replication 44, 45. Like previous sequencing techniques based on step‐by‐step lengthening of sequencing and labeling of the final nucleotide, Sanger sequencing outperformed them all due to the precision, robustness and usability of the chain‐termination method, which made the Sanger technique the most widely used method in more than two and a half decades. Automation of the Sanger sequencing method became a reality in 1986 when Applied Biosystems launched the automated DNA sequencing instrument where, although still based on the Sanger method, each nucleotide was now labeled with a fluorescent dye and allowing a read out of specific nucleotides by color 46. Also, the Applied sequencing instrument enabled 24 samples to run at the same time and generated sequencing data faster and cheaper than ever before. This automated Sanger technique, also known as ‘first‐generation sequencing’, allowed one of the largest projects in human science; ‘The Human Genome project’. The Human Genome project set out to sequence every human chromosome with the objective to advance knowledge of human biology and improve medicine. After years of planning, the project was formally launched in 1990, and after a 13‐year‐long endeavor the project completed in 2003, 2 years ahead of schedule 47, 48. It still remains the world's largest collaborative biological project with a budget of $3 billion 49.

Following the complete sequencing and mapping of the 24 human chromosomes, the demand for sequencing increased rapidly, thus calling for a more low‐cost and high‐throughput technique. This calling motivated the development of second‐generation sequencing, also known as next‐generation sequencing (NGS), where pyrosequencing plays a major role. The pyrosequencing technique, pioneered by Pål Nyren and colleagues 50, 51 was still, like the Sanger's dideoxy, a sequence‐by synthesis’ (SBS) technique, as they both require a direct action of DNA polymerase to produce the reading output. The pyrosequencing method is based on detecting the activity of the DNA polymerase with luminescence, hence allowing sequencing a single strand of DNA by synthesizing the complementary strand, one base pair at a time. Each nucleotide is sequentially washed through the system over the template DNA affixed to a solid phase. Light is produced only when the nucleotide solution complements the first unpaired base of the template and the order of solutions which produce the luminescence permits the determination of the sequence of the template. The first major successful commercial NGS platform, the 454 sequencer was based on the concept of pyrosequencing technique and mass parallelization of sequencing reactions, greatly increasing the amount of DNA that can be sequenced in one run 52. Several parallel sequencing techniques followed upon the success of the 454 sequencer where the Solexa method of sequencing, acquired by Illumina in 2006, is the most important and ‘short read’ sequencing platform 53. In short, template DNA is fragmented and end‐repaired with poly‐A tails to ensure the ligation of adaptors. The adapter oligonucleotides are complementary to the flow‐cell anchors. Adapter‐modified, single‐stranded DNA is added to the flow cell and immobilized by hybridization. Bridge amplification generates clonally amplified clusters that are denatured and cleaved, and sequencing can be initiated with addition of primers, polymerase and the four reversible dye terminators, followed by imaging and recording of fluorescence. When recording of the one cycle is completed, fluorescence and terminators are removed, and the next cycle of synthesis is directly initiated.

Targeted sequencing and gene‐panels are widely used when predefined genes are to be sequenced and if coverage need to be high, for instance to identify low‐frequency tumor‐drives. The actual sequencing process is identical to the one described above, however the region of interest is captured by initial hybridization of a library of biotinylated RNA oligos predesigned to specific genomic regions (e.g. gene‐panel) using magnetic beads, flowed by PCR amplification. It is often argued that targeted sequencing is preferable if the suspected disease or condition has already been identified, due to affordable costs and higher coverage yield as well as reduced sequencing time 54. However, it may be reasonable to question this presumption, since it holds some frauds; for instance, sequencing is often used to confirm a tentative diagnosis, and often, variants in the suspected genes associated with the most likely diagnosis are not identified, resulting in another targeted gene panel or a broader screening method like WES or WGS. Thus, choosing targeted gene‐panel may indeed increase the time until a causative genetic diagnosis is made, as well as the costs, which would have been reduced if the genomic approach had been the first and only platform chosen 55, 56, 57. Other arguments for working towards reducing the use of gene‐panels and convert to WGS as the primary choice, is that the sequencing data can be remapped and reanalyzed at a later stage, when new genetic associations are identified; moreover, the data together with the patients phenotype enables research in a specific disease and will lead to the identification of new disease associated candidate genes without further laboratory costs. Interestingly, when comparing sample preparation time in the laboratory, the pre‐capture‐step necessary for gene‐panels exceeds the non‐capture technique used for WGS, both in time and hands‐on 58.

**Figure 1:**
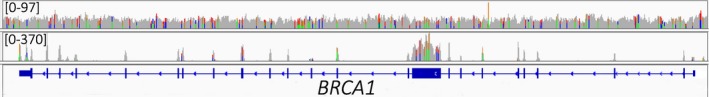
*BRCA1* gene sequence alignments from the same patient by WGS (top) and WES (bottom). The coverage span of the entire genome in this sample's gene was from 0–97 and the entire exome 0–370. WES, whole‐exome sequencing; WGS, whole‐genome sequencing.

Whole‐exome‐sequencing is also a result of a pre‐capture‐step, this time of all coding exons of the genome, allowing a sequential screen of the exome. In contrast, WGS does not include a capturing step, since it is the entire genome that is intended for sequencing; both coding and non‐coding. This fundamental difference which may best be illustrated by a raw sequence output (Fig. 1), greatly affects the downstream applications. WGS provides an incomparable complete coverage of the exome thus, WGS is simply the better WES 59. As such, and with costs declining and with the appropriate *in silico* panels, WGS has the potential to entirely replace WES and other techniques that involves capturing of target sequences. In addition, a wide range of WGS data applications, like insertion‐deletions, copy‐number variation (CNV), intronic deletions, structural variation and repetitive DNA element, substantiates why this sequencing platform is superior to WES. It is so far not possible to interpret the significance of most of the additional findings from WGS but then only through continuous sequencing studies, the knowledge database will expand and enable implementation of precision medicine in a clinical setting. However, since average coverage of a WGS is around 30–40×, WGS is not optimal for identification of mosaicism or other low‐frequent variants e.g. tumor‐drivers. For these purposes, a targeted gene‐panel with a minimum of 500× is more suitable. The cost of a WGS opposed to WES and gene‐panels is a major concern. The price of sequencing is constantly declining and at present time a WGS costs less than $1.5K 60. The need for data storage, curation and bioinformatic processing is substantial if WGS‐analysis is implemented as a routine genetic test 61, 62. Consequently, most western countries are launching national initiatives for large‐scale whole‐genome‐sequencing projects where essential computer‐ and man‐power is centered. Obviously, the long‐term impact on health economics is not yet understood. Discussions about how to manage the risk of identifying incidental genomic findings have emerged as one of the more contentious issues in the clinical application of genomic sequencing 63, 64. The subject is to vast to review in the present context, however the key elements that the discussions has brought is the transition of *incidental* to *secondary* findings and the patients right to ‘option out’ on secondary findings. The American College of Medical Genetics and Genomics have recently updated their recommendations on how to address secondary findings in a clinical setting, hereby enabling consensus amongst sequencing laboratories 65. The basic laboratory workflow and downstream data pipeline and various applications of WGS will be addressed in the following.

**Figure 2:**
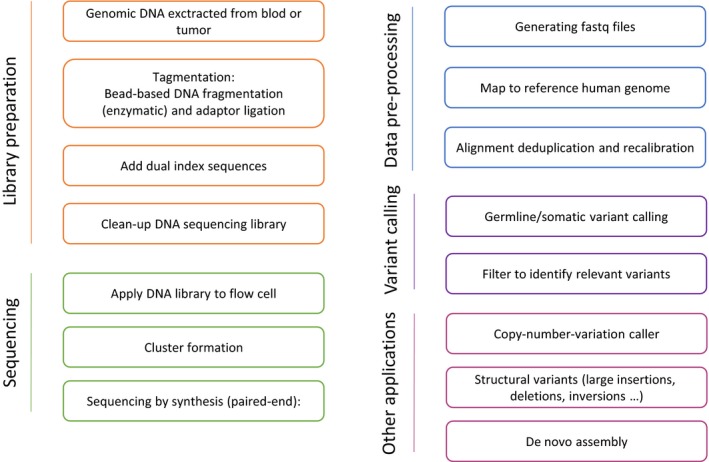
Whole‐genome‐sequencing workflow and bioinformatic pipeline. The illustrated workflow is based on the Illumina WGS platform. WGS, whole‐genome sequencing.

An overview of the laboratory workflow for generating WGS sequencing results is shown in Fig. 2 and is described only for the commonly applied Illumina protocol. Genomic DNA purified from either whole blood or tumor is prepared for library by using Nextera DNA Flex Library Preparation Kit with dual indexes. In brief, 10–500 ng of genomic DNA is used as input for the on‐bead tagmentation step, followed by a limited PCR amplification and bead clean‐up of the WGS DNA sequencing library. Sequencing can be readily performed as 2 × 150 bp paired‐end sequencing on a NovaSeq6000 instrument. Raw fastq files are mapped to the hg19/GRCh37 human reference genome using BWA‐MEM v0.7.12 (Li, 2013) software. Quality thresholds for the sequencing are >30‐fold average sequencing depth and >98% of the genome sequenced at least 10‐fold. Alignment file pre‐processing and germline variant calling is performed by GATK v3.8.0 using Best Practices guidelines 66. For clinical implications and from sample to clinical reporting, the entire laboratory workflow and data processing can be completed in less than a week, depending on the downstream manual and medical variant classification. Preprocessed WGS data can be used to identify for structural variants e.g. CNVs and fusion genes or CNVs 59, 67, 68. Furthermore, WGS data enables *de novo* assembly, which is an alternative way of variant calling, since it does not map to a reference genome, in other words, the variant caller does not assume any normal positions; thus, *de novo* assembly allows ‘wrong’ reads which may reflect insertions, inversions or translocations 69, 70. It should be noted, that if DNA originates from tumor tissue the standard algorithm for data processing and variant calling does not explicit consider tumor impurity or intra‐tumor‐heterogeneity. So far, estimation of tumor content and identification of clonal somatic aberrations require high‐coverage sequencing, which is not the case for WGS data where coverage is limited (30–40×). Comparative studies clearly show that tolls developed for high‐sequence coverage data are not suitable for WGS data 71. Accordingly, bioinformatic tools for deconvolution of genomic low‐coverage data are emerging 61, 72.

## Hereditary breast cancer

One out of every seven women diagnosed with invasive breast cancer will have a close relative (mother, sister, or daughter) also diagnosed with the disease. Pathogenic variants in the two major breast cancer susceptibility genes *BRCA1* and *BRCA2* may explain 15% of increased risk of breast cancer among female relatives of breast cancer patients. Germline variants in several other susceptibility genes also confer a high risk of breast cancer, including pathogenic variants in *ATM, BARD1, BLM, BRIP1, CDH1, CHEK2, PALB2, PMS2, FAM175A, FANCC/‐M,* and *RAD51B/‐C/-D* as well as the more syndromic predisposing genes; *TP53* (Li–Fraumeni syndrome), *PTEN* (Cowdens disease), *STK11* (Peutz‐Jeghers syndrome), *NF1* (Neurofibromatosis), and *CDH1* (Hereditary diffuse gastric cancer syndrome). Large panels of breast cancer susceptibility genes have become widely available 73. Identification of pathogenic *BRCA1* and *BRCA2* variants is routinely used to predict risk of breast‐ and ovarian cancer and guide the use of risk reducing surgery, thus upfront genetic screening of all new breast cancer patients enables a more tailored surgical procedure 74, 75, 76, 77. Hence, the meta‐analysis by Li et al., recently substantiated the significant decrease in overall mortality for patients with pathogenic *BRCA1* or *BRCA2* variants undergoing contralateral prophylactic mastectomy as part of the standard breast cancer intervention. Adjuvant chemotherapy is not recommended to all patients with ER‐positive and HER2‐normal breast cancer but may in women with germline pathogenic *BRCA1* or *BRCA2* variants confer a distinct survival benefit 75, 78. Furthermore, platinum‐based chemotherapy has in the neoadjuvant and metastatic setting been superior to conventional anthracycline and taxane based regimens 79. Finally, targeting impaired BRCA1 or BRCA2 by poly ADP ribose polymerase‐inhibitors (PARPi) is approved for metastatic breast cancer in women with germline pathogenic *BRCA1* or *BRCA2* variants 80, 81. Accordingly, up‐front screening for *BRCA1* and *BRCA2* variants is increasingly offered to patients at the diagnosis of breast cancer and may be completed in less than a week 41. National consensus on breast cancer panel testing is emerging and may result in clinical utility of a wider range of germline variants 82. It is evident, that more breast cancer predisposing genes will occur and for this purpose, an international screening program was established to assemble the sequencing data and collaborating on identifying and validating new candidate breast cancer genes 83.

## Breast cancer and genome maintenance

**Figure 3:**
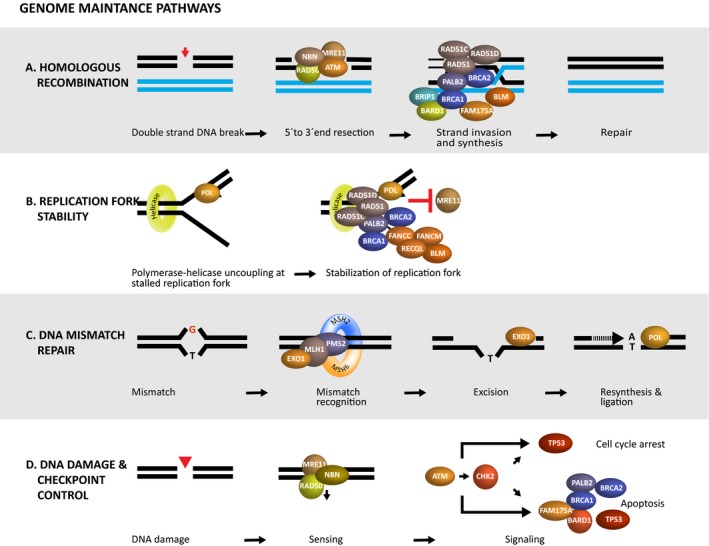
Major genome stability pathways affected in hereditary breast cancer. (A) Homologous recombination repair (HRR) is initiated by DNA end resection which removes the 5′‐strand leaving a 3′‐single‐stranded DNA (ssDNA) strand. This process is promoted by ataxia telangiectasia mutated (ATM) and the MRN complex where MRE11 is the nuclease. BRCA2 and PALB2 then load RAD51 onto the resected 3′‐ssDNA strand, forming the nucleoprotein filament. RAD51 mediates pairing with the complementary DNA sequence on the sister chromatid, used as template for DNA synthesis. HRR is concluded by ligation of the DNA ends. (B) Replication fork protection secures DNA at stalling forks from nuclease‐driven degradation of the newly synthesized DNA. HBOC factors limit degradation mainly by counteracting MRE11 nuclease activity. (C) DNA Mismatch repair promotes genome stability through correction of erroneous DNA base pairing occurring following DNA replication or DNA repair synthesis. MLH1, MSH2 and PMS2 are HBOC factors. (D) DNA damage and checkpoint control respond to DNA lesions by direct cell fate options. The MRN complex is thought to recruit ATM via NBN, thereby initiating kinase cascades together with its downstream target CHK2. This suppresses cell cycle progression in G1, S and G2 phases of the cell cycle, allowing time for DNA repair. Following marked unrepaired damage, cell death pathways can be activated to eradicate cells with marked potential for dysfunction.

Pathogenic variants in *BRCA1* and *BRCA2* predispose to hereditary breast and ovarian cancer (HBOC), but only 15–25% of HBOC cases can be ascribed to either of the genes. Recently, exome sequencing has uncovered a substantial locus heterogeneity among affected families without *BRCA1* and *BRCA2* pathogenic variants and in the same way as *BRCA1* and *BRCA2* the novel putative HBOC susceptibility genes are involved in genome stability pathways such as homologous recombination repair, and to a minor extent mismatch repair (MMR) and interstrand cross‐link repair, Fig. 3 84. DNA double‐strand breaks occurring during DNA replication are frequently corrected by homologous recombination repair (HRR). In principle, HRR is an error‐free DNA repair pathway, because it employs the intact sister chromatid as template for the repair reaction. The DNA double‐stranded ends cannot be used in HRR, because such DNA cannot align with the correct corresponding DNA sequence of the sister chromatid. Thus, the DNA ends are processed to single‐stranded DNA ends before BRCA2 loads RAD51 onto the ssDNA end. The RAD51‐coated DNA end then searches and anneals with the corresponding sister chromatid DNA sequence 85. Whereas BRCA2 has a very well‐defined role in HRR with RAD51, the role of BRCA1 is not entirely understood. BRCA1 interacts with a wide range of proteins and may simply function as recruitment scaffold. Moreover, BRCA1 haploinsufficiency may lead to an inadequate response to replication stress, thereby increasing the risk of cancer 86.

Additional factors such as PALB2, ATM, BARD1, BRIP1, FAM175A, NBN, MRE11, BLM, and RAD51 paralogs (RAD51B, RAD51C, RAD51D, XRCC2, and XRCC3) are also necessary for HRR 84, 85, 86. PALB2 stabilizes BRCA2, and RAD51 paralogs are important for formation of functional RAD51‐ssDNA ends 85. BARD1 heterodimerizes with BRCA1 which has a protein stabilizing impact on BRCA1 87. *BARD1*,* PALB2,* and *RAD51B* variants have been identified in breast cancer families, whereas truncating *RAD51C* and *RAD51D* variants are mainly found in families with ovarian cancer or breast and ovarian cancer. BRIP1 and FAM175A are both BRCA1 interacting proteins involved in recruitment of BRCA1 to DNA DSBs 84. The *BRIP1* gene was originally suggested to be a low‐penetrant breast cancer susceptibility gene, but, later studies suggested that the risk was elevated for ovarian cancer. Pathogenic variants in *FAM175A* (Abraxas) have been identified in both breast and ovarian cancer patients, however, currently the lifetime risk for breast‐ and ovarian cancer is unknown 84. The Mre11‐Rad50‐Nbs1 (MRN) complex is also necessary for detection and signaling of DNA DSBs 88. So far, only *MRE11* and *NBN* variants have been clinically associated to breast and ovarian cancer. The MRN complex activates the ATM (Ataxia Telangiectasia‐Mutated) serine/threonine protein kinase that phosphorylates factors in the DNA damage response including TP53, CHK2, and CtIP (*RBBP8*) 89. A number of rare HBOC factors such as BLM, RECQL, FANCC, and FANCM may also contribute to HBOC by protecting DNA replication forks and suppressing DNA replication stress 84.

Amongst the DNA repair pathways, mismatch repair (MMR) is also involved though less frequently than HRR. The MMR genes *MLH1*,* MSH2*,* MSH6,* and *PMS2* that were originally implicated in Lynch syndrome or hereditary nonpolyposis colorectal cancer (HNPCC), also play a role in breast and ovarian cancer since haploinsufficiency of *MLH1*,* MSH2,* and *PMS2* appears to increase the risk for ovarian cancer and to a minor extent breast cancer 90, 91. The MMR system recognizes and repairs misincorporation of nucleotides and defects in MMR results in the accumulation of thousands of single nucleotide variants and microsatellite instability (MSI) in the tumors 92. Tumor suppressor genes, such as *MRE11* and *RAD50* genes, moreover, harbor microsatellites so impaired MMR may indirectly affect the MRN complex 93.

DNA repair is undoubtedly important in suppressing HBOC, but nevertheless, additional pathways related to genome stability and DNA damage response are also relevant. In this regard, cell cycle checkpoints and cell death pathways may also be implicated in HBOC. These pathways normally eliminate cells with damaged DNA and haploinsufficiency of check‐point regulators genes leads to accumulation of mutated cells. The archetypical check‐point regulator is TP53 which coordinates several genome stability factors. ATM, CHK2 (encoded by *CHEK2*), TP53, BRCA1–BARD1 can block the cell cycle in G1, S and in G2 phase checkpoints 94, 95. Constitutive loss of TP53 leads to the autosomal dominant Li–Fraumeni syndrome (LFS) with breast cancer, sarcoma, brain tumors, and adrenocortical carcinoma 96, 97. TP53 is activated by the ATM kinase that phosphorylates CHK2, and in an amplifying step CHK2 subsequently further targets TP53 thereby promoting the actions of ATM 89, 98. The clinical significance of the mechanism is illustrated by the *CHEK2* c.1100delC variant that is associated with approximately three‐fold increased risk of breast cancer 99, 100. Taken together, cells with impaired DNA repair and cell cycle checkpoints are ultimately likely to gain selective proliferative advantages.

The recent progress in next generation sequencing makes it possible to identify predisposing genetic variants in a fast and cost‐effective way. We, however, face a situation where cohort and co‐segregation data may not be available for rare variants, and it is important to investigate at the protein level, for example, by employing structural and functional analysis for variant classification. Since the extensive locus heterogeneity appears to converge on a relatively small number of genome maintenance pathways, we predict that such analysis may become a reality within a relatively short period of time. Unfounded classification of genetic variants is obviously harmful to the patient, and great care should be taken to generate common protocols and accreditation of the analysis to meet clinical standards.

**Figure 4:**
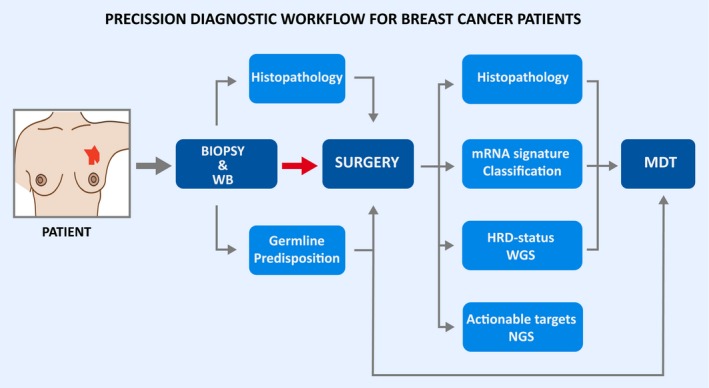
Precision diagnostic workflow for breast cancer patients; Once a diagnosis of breast cancer has been verified from core‐needle biopsy, a germline analysis by whole‐genome sequencing (WGS) is initiated from whole‐blood (WH). Initially, genetic status of the major predisposing genes should be ready in time for planning of risk reducing surgery. Based on DNA and RNA from the surgical specimen, a full somatic profile and subtype is generated and finalized for multidisciplinary team (MDT) including; HRD‐status and mutation burden from DNA analyzed by WGS (PARPi or immunotherapy) as well as potential actionable targets from high‐coverage NGS‐panel; Molecular subtype, receptor status and proliferative index from RNA based on RNA‐sequencing. Histopathology includes immunohistochemical type, morphological characteristics and receptor status.

Whole‐genome sequencing can be used to detect an impairment in two of the major pathways in the genome‐maintenance system; HRR and MMR. For detection of homolog recombination deficiency (HRD) by WGS, Davies et al. recently generated a predictor for BRCA1 and BRCA2‐defecient tumor samples. This WGS‐based predictor may be well‐suited for the clinical setting since the study showed a doubling in the detection rate of HRD tumors as well as robustness between independent biopsies and samples, including DNA obtained from paraffin‐embedded tissue 101. The signature named *HRDetect* includes base substitutions, indels and rearrangements of many more genes than just *BRCA1* and *BRCA2* and is trained to suite WGS derived results. Identifying tumors that are deficient in HRR, whether it is through germline or somatic inactivation of BRCA1 and BRCA2 is clinically extremely relevant since these tumors are selectively sensitive to PARPi 102, 103. For identification of MMR‐impaired breast cancer tumors, another WGS based mutational signature has recently been described 104. The mutational signature is sought to be a direct pathophysiological reflection of MMR pathway abrogation that may outperform current biomarkers and hereby increasing the sensitivity to immune therapies; Thus, WGS should soon be required for up‐front routine clinical analysis and we suggest an optimized diagnostic workflow which allows the performance of precision medicine, Fig. 4.

## References

[1] Hyman DM , Taylor BS , Baselga J . Implementing genome‐driven oncology. Cell 2017;168:584–99. 28187282 10.1016/j.cell.2016.12.015 PMC5463457

[2] Comprehensive molecular portraits of human breast tumours. Nature 2012;490:61–70. 23000897 10.1038/nature11412 PMC3465532

[3] Kandoth C , McLellan MD , Vandin F , Ye K , Niu B , Lu C , et al. Mutational landscape and significance across 12 major cancer types. Nature 2013;502:333. 24132290 10.1038/nature12634 PMC3927368

[4] Weinstein JN , Collisson EA , Mills GB , Shaw KR , Ozenberger BA , Ellrott K , et al. The cancer genome atlas pan‐cancer analysis project. Nat Genet 2013;45:1113–20. 24071849 10.1038/ng.2764 PMC3919969

[5] Berger MF , Mardis ER . The emerging clinical relevance of genomics in cancer medicine. Nat Rev Clin Oncol 2018;15:353–65. 29599476 10.1038/s41571-018-0002-6 PMC6658089

[6] Kumar‐Sinha C , Chinnaiyan AM . Precision oncology in the age of integrative genomics. Nat Biotechnol 2018;36:46. 29319699 10.1038/nbt.4017 PMC6364676

[7] Slamon DJ , Leyland‐Jones B , Shak S , Fuchs H , Paton V , Bajamonde A , et al. Use of chemotherapy plus a monoclonal antibody against HER2 for metastatic breast cancer that overexpresses HER2. N Engl J Med 2001;344:783–92. 11248153 10.1056/NEJM200103153441101

[8] Yates LR , Knappskog S , Wedge D , Farmery JHR , Gonzalez S , Martincorena I , et al. Genomic evolution of breast cancer metastasis and relapse. Cancer Cell 2017;32:169–84.e7. 28810143 10.1016/j.ccell.2017.07.005 PMC5559645

[9] Siegel RL , Miller KD , Jemal A . Cancer statistics, 2017. CA Cancer J Clin 2017;67:7–30. 28055103 10.3322/caac.21387

[10] Engholm G , Ferlay J , Christensen N , Hansen HL , Hertzum‐Larsen R , Johannesen TB , et al. NORDCAN: Cancer Incidence, Mortality, Prevalence and Survival in the Nordic Countries, Version 8.0 http://www.ancr.nu: Association of the Nordic Cancer Registries. Danish Cancer Society; 2017.

[11] Harbeck N , Gnant M . Breast cancer. Lancet 2017;389:1134–50. 27865536 10.1016/S0140-6736(16)31891-8

[12] Candido Dos Reis FJ , Wishart GC , Dicks EM , Greenberg D , Rashbass J , Schmidt MK , et al. An updated PREDICT breast cancer prognostication and treatment benefit prediction model with independent validation. Breast Cancer Res 2017;19:58. 28532503 10.1186/s13058-017-0852-3 PMC5440946

[13] Ejlertsen B , Jensen MB , Mouridsen HT . Excess mortality in postmenopausal high‐risk women who only receive adjuvant endocrine therapy for estrogen receptor positive breast cancer. Acta Oncol 2014;53:174–85. 24219541 10.3109/0284186X.2013.850738

[14] McLaughlin SA . Surgical management of the breast: breast conservation therapy and mastectomy. Surg Clin North Am 2013;93:411–28. 23464693 10.1016/j.suc.2012.12.006

[15] Goldhirsch A , Winer EP , Coates AS , Gelber RD , Piccart‐Gebhart M , Thurlimann B , et al. Personalizing the treatment of women with early breast cancer: highlights of the St Gallen International Expert Consensus on the Primary Therapy of Early Breast Cancer 2013. Ann Oncol 2013;24:2206–23. 23917950 10.1093/annonc/mdt303 PMC3755334

[16] Clough KB , Acosta‐Marin V , Nos C , Alran S , Rouanet P , Garbay JR , et al. Rates of neoadjuvant chemotherapy and oncoplastic surgery for breast cancer surgery: a French National Survey. Ann Surg Oncol 2015;22:3504–11. 25665949 10.1245/s10434-015-4378-6

[17] Mougalian SS , Soulos PR , Killelea BK , Lannin DR , Abu‐Khalaf MM , DiGiovanna MP , et al. Use of neoadjuvant chemotherapy for patients with stage I to III breast cancer in the United States. Cancer 2015;121:2544–52. 25902916 10.1002/cncr.29348

[18] Early Breast Cancer Trialists’ Collaborative Group (EBCTCG) , Asselain B , Barlow W , Bartlett J , Bergh J , Bergsten‐Nordström E , et al.Long‐term outcomes for neoadjuvant versus adjuvant chemotherapy in early breast cancer: meta‐analysis of individual patient data from ten randomised trials. Lancet Oncol 2018;19:27–39. 29242041 10.1016/S1470-2045(17)30777-5 PMC5757427

[19] Cortazar P , Zhang L , Untch M , Mehta K , Costantino JP , Wolmark N , et al. Pathological complete response and long‐term clinical benefit in breast cancer: the CTNeoBC pooled analysis. Lancet 2014;384:164–72. 24529560 10.1016/S0140-6736(13)62422-8

[20] Foulkes WD , Stefansson IM , Chappuis PO , Begin LR , Goffin JR , Wong N , et al. Germline BRCA1 mutations and a basal epithelial phenotype in breast cancer. J Natl Cancer Inst 2003;95:1482–5. 14519755 10.1093/jnci/djg050

[21] Perou CM , Sorlie T , Eisen MB , van de Rijn M , Jeffrey SS , Rees CA , et al. Molecular portraits of human breast tumours. Nature 2000;406:747–52. 10963602 10.1038/35021093

[22] Sorlie T , Perou CM , Tibshirani R , Aas T , Geisler S , Johnsen H , et al. Gene expression patterns of breast carcinomas distinguish tumor subclasses with clinical implications. Proc Natl Acad Sci USA 2001;98:10869–74. 11553815 10.1073/pnas.191367098 PMC58566

[23] Sorlie T , Tibshirani R , Parker J , Hastie T , Marron JS , Nobel A , et al. Repeated observation of breast tumor subtypes in independent gene expression data sets. Proc Natl Acad Sci USA 2003;100:8418–23. 12829800 10.1073/pnas.0932692100 PMC166244

[24] Sotiriou C , Neo SY , McShane LM , Korn EL , Long PM , Jazaeri A , et al. Breast cancer classification and prognosis based on gene expression profiles from a population‐based study. Proc Natl Acad Sci USA 2003;100:10393–8. 12917485 10.1073/pnas.1732912100 PMC193572

[25] van ‘t Veer LJ , Dai H , van de Vijver MJ , He YD , Hart AA , Mao M , et al. Gene expression profiling predicts clinical outcome of breast cancer. Nature 2002;415:530–6. 11823860 10.1038/415530a

[26] Parker JS , Mullins M , Cheang MC , Leung S , Voduc D , Vickery T , et al. Supervised risk predictor of breast cancer based on intrinsic subtypes. J Clin Oncol 2009;27:1160–7. 19204204 10.1200/JCO.2008.18.1370 PMC2667820

[27] Cardoso F , van't Veer LJ , Bogaerts J , Slaets L , Viale G , Delaloge S , et al. 70‐Gene signature as an aid to treatment decisions in early‐stage breast cancer. N Engl J Med 2016;375:717–29. 27557300 10.1056/NEJMoa1602253

[28] Sparano JA , Gray RJ , Makower DF , Pritchard KI , Albain KS , Hayes DF , et al. Adjuvant chemotherapy guided by a 21‐gene expression assay in breast cancer. N Engl J Med 2018;379:111–21. 29860917 10.1056/NEJMoa1804710 PMC6172658

[29] Curtis C , Shah SP , Chin SF , Turashvili G , Rueda OM , Dunning MJ , et al. The genomic and transcriptomic architecture of 2,000 breast tumours reveals novel subgroups. Nature 2012;486:346–52. 22522925 10.1038/nature10983 PMC3440846

[30] Guedj M , Marisa L , de Reynies A , Orsetti B , Schiappa R , Bibeau F , et al. A refined molecular taxonomy of breast cancer. Oncogene 2012;31:1196–206. 21785460 10.1038/onc.2011.301 PMC3307061

[31] Nik‐Zainal S , Davies H , Staaf J , Ramakrishna M , Glodzik D , Zou X , et al. Landscape of somatic mutations in 560 breast cancer whole‐genome sequences. Nature 2016;534:47–54. 27135926 10.1038/nature17676 PMC4910866

[32] Burstein MD , Tsimelzon A , Poage GM , Covington KR , Contreras A , Fuqua SA , et al. Comprehensive genomic analysis identifies novel subtypes and targets of triple‐negative breast cancer. Clin Cancer Res 2015;21:1688–98. 25208879 10.1158/1078-0432.CCR-14-0432 PMC4362882

[33] Chen X , Li J , Gray WH , Lehmann BD , Bauer JA , Shyr Y , et al. TNBCtype: a subtyping tool for triple‐negative breast cancer. Cancer Inform 2012;11:147–56. 22872785 10.4137/CIN.S9983 PMC3412597

[34] Gatza ML , Lucas JE , Barry WT , Kim JW , Wang Q , Crawford MD , et al. A pathway‐based classification of human breast cancer. Proc Natl Acad Sci USA 2010;107:6994–9. 20335537 10.1073/pnas.0912708107 PMC2872436

[35] Jezequel P , Loussouarn D , Guerin‐Charbonnel C , Campion L , Vanier A , Gouraud W , et al. Gene‐expression molecular subtyping of triple‐negative breast cancer tumours: importance of immune response. Breast Cancer Res 2015;17:43. 25887482 10.1186/s13058-015-0550-y PMC4389408

[36] Lehmann BD , Bauer JA , Chen X , Sanders ME , Chakravarthy AB , Shyr Y , et al. Identification of human triple‐negative breast cancer subtypes and preclinical models for selection of targeted therapies. J Clin Investig 2011;121:2750–67. 21633166 10.1172/JCI45014 PMC3127435

[37] Prat A , Pineda E , Adamo B , Galvan P , Fernandez A , Gaba L , et al. Clinical implications of the intrinsic molecular subtypes of breast cancer. Breast 2015;24(Suppl 2):S26–35. 26253814 10.1016/j.breast.2015.07.008

[38] Coates AS , Winer EP , Goldhirsch A , Gelber RD , Gnant M , Piccart‐Gebhart M , et al. Tailoring therapies–improving the management of early breast cancer: St Gallen International Expert Consensus on the Primary Therapy of Early Breast Cancer 2015. Ann Oncol 2015;26:1533–46. 25939896 10.1093/annonc/mdv221 PMC4511219

[39] Prat A , Cheang MC , Martin M , Parker JS , Carrasco E , Caballero R , et al. Prognostic significance of progesterone receptor‐positive tumor cells within immunohistochemically defined luminal A breast cancer. J Clin Oncol 2013;31:203–9. 23233704 10.1200/JCO.2012.43.4134 PMC3532392

[40] Viale G , Regan MM , Maiorano E , Mastropasqua MG , Dell'Orto P , Rasmussen BB , et al. Prognostic and predictive value of centrally reviewed expression of estrogen and progesterone receptors in a randomized trial comparing letrozole and tamoxifen adjuvant therapy for postmenopausal early breast cancer: BIG 1‐98. J Clin Oncol 2007;25:3846–52. 17679725 10.1200/JCO.2007.11.9453

[41] Rossing M , Ostrup O , Majewski WW , Kinalis S , Jensen MB , Knoop A , et al. Molecular subtyping of breast cancer improves identification of both high and low risk patients. Acta Oncol 2018;57:58–66. 29164972 10.1080/0284186X.2017.1398416

[42] F. S. Frederick Sanger — Biographical 1980. Available from: http://www.nobelprize.org/nobel_prizes/chemistry/laureates/1980/sanger-bio.html

[43] Watson JD , Crick FH . Molecular structure of nucleic acids: a structure for deoxyribose nucleic acid. Nature 1974;248:765. 4599080 10.1038/248765a0

[44] Sanger F , Coulson AR . A rapid method for determining sequences in DNA by primed synthesis with DNA polymerase. J Mol Biol 1975;94:441–8. 1100841 10.1016/0022-2836(75)90213-2

[45] Sanger F , Nicklen S , Coulson AR . DNA sequencing with chain‐terminating inhibitors. Proc Natl Acad Sci USA 1977;74:5463–7. 271968 10.1073/pnas.74.12.5463 PMC431765

[46] Smith LM , Sanders JZ , Kaiser RJ , Hughes P , Dodd C , Connell CR , et al. Fluorescence detection in automated DNA sequence analysis. Nature 1986;321:674–9. 3713851 10.1038/321674a0

[47] Lander ES , Linton LM , Birren B , Nusbaum C , Zody MC , Baldwin J , et al. Initial sequencing and analysis of the human genome. Nature 2001;409:860–921. 11237011 10.1038/35057062

[48] Venter JC , Adams MD , Myers EW , Li PW , Mural RJ , Sutton GG , et al. The sequence of the human genome. Science 2001;291:1304–51. 11181995 10.1126/science.1058040

[49] Tripp S . GM. Economic Impact of the Human Genome Project – Battelle 2011. Available from: https://www.battelle.org/docs/default-source/misc/battelle-2011-misc-economic-impact-human-genome-project.pdf

[50] Nyren P , Pettersson B , Uhlen M . Solid phase DNA minisequencing by an enzymatic luminometric inorganic pyrophosphate detection assay. Anal Biochem 1993;208:171–5. 8382019 10.1006/abio.1993.1024

[51] Ronaghi M , Karamohamed S , Pettersson B , Uhlen M , Nyren P . Real‐time DNA sequencing using detection of pyrophosphate release. Anal Biochem 1996;242:84–9. 8923969 10.1006/abio.1996.0432

[52] Margulies M , Egholm M , Altman WE , Attiya S , Bader JS , Bemben LA , et al. Genome sequencing in microfabricated high‐density picolitre reactors. Nature 2005;437:376–80. 16056220 10.1038/nature03959 PMC1464427

[53] Voelkerding KV , Dames SA , Durtschi JD . Next‐generation sequencing: from basic research to diagnostics. Clin Chem 2009;55:641–58. 19246620 10.1373/clinchem.2008.112789

[54] Xuan J , Yu Y , Qing T , Guo L , Shi L . Next‐generation sequencing in the clinic: promises and challenges. Cancer Lett 2013;340:284–95. 23174106 10.1016/j.canlet.2012.11.025 PMC5739311

[55] Lee H , Deignan JL , Dorrani N , Strom SP , Kantarci S , Quintero‐Rivera F , et al. Clinical exome sequencing for genetic identification of rare Mendelian disorders. JAMA 2014;312:1880–7. 25326637 10.1001/jama.2014.14604 PMC4278636

[56] Valencia CA , Husami A , Holle J , Johnson JA , Qian Y , Mathur A , et al. Clinical impact and cost‐effectiveness of whole exome sequencing as a diagnostic tool: a pediatric center's experience. Front Pediatr 2015;3:67. 26284228 10.3389/fped.2015.00067 PMC4522872

[57] Shashi V , McConkie‐Rosell A , Rosell B , Schoch K , Vellore K , McDonald M , et al. The utility of the traditional medical genetics diagnostic evaluation in the context of next‐generation sequencing for undiagnosed genetic disorders. Genet Med 2014;16:176–82. 23928913 10.1038/gim.2013.99

[58] Stranneheim H , Engvall M , Naess K , Lesko N , Larsson P , Dahlberg M , et al. Rapid pulsed whole genome sequencing for comprehensive acute diagnostics of inborn errors of metabolism. BMC Genom 2014;15:1090. 10.1186/1471-2164-15-1090 PMC4299811 25495354

[59] Meienberg J , Bruggmann R , Oexle K , Matyas G . Clinical sequencing: is WGS the better WES? Hum Genet 2016;135:359–62. 26742503 10.1007/s00439-015-1631-9 PMC4757617

[60] Wetterstrand KA . DNA Sequencing Costs: Data from the NHGRI Genome Sequencing Program (GSP). 2014 Available from: http://www.genome.gov/sequencingcosts

[61] He KY , Ge D , He MM . Big data analytics for genomic medicine. Int J Mol Sci 2017;18:412. 10.3390/ijms18020412 PMC5343946 28212287

[62] Oakeson KF , Wagner JM , Mendenhall M , Rohrwasser A , Atkinson‐Dunn R . Bioinformatic analyses of whole‐genome sequence data in a public health laboratory. Emerg Infect Dis 2017;23:1441–5. 28820135 10.3201/eid2309.170416 PMC5572866

[63] Berg JS , Khoury MJ , Evans JP . Deploying whole genome sequencing in clinical practice and public health: meeting the challenge one bin at a time. Genet Med 2011;13:499. 21558861 10.1097/GIM.0b013e318220aaba

[64] Roche MI , Berg JS . Incidental findings with genomic testing: implications for genetic counseling practice. Curr Genet Med Rep 2015;3:166–76. 26566463 10.1007/s40142-015-0075-9 PMC4633435

[65] Kalia SS , Adelman K , Bale SJ , Chung WK , Eng C , Evans JP , et al. Recommendations for reporting of secondary findings in clinical exome and genome sequencing, 2016 update (ACMG SF v2.0): a policy statement of the American College of Medical Genetics and Genomics. Genet Med 2017;19:249–55. 27854360 10.1038/gim.2016.190

[66] McKenna A , Hanna M , Banks E , Sivachenko A , Cibulskis K , Kernytsky A , et al. The Genome Analysis Toolkit: a MapReduce framework for analyzing next‐generation DNA sequencing data. Genome Res 2010;20:1297–303. 20644199 10.1101/gr.107524.110 PMC2928508

[67] Abyzov A , Urban AE , Snyder M , Gerstein M . CNVnator: an approach to discover, genotype, and characterize typical and atypical CNVs from family and population genome sequencing. Genome Res 2011;21:974–84. 21324876 10.1101/gr.114876.110 PMC3106330

[68] Chen X , Schulz‐Trieglaff O , Shaw R , Barnes B , Schlesinger F , Kallberg M , et al. Manta: rapid detection of structural variants and indels for germline and cancer sequencing applications. Bioinformatics 2016;32:1220–2. 26647377 10.1093/bioinformatics/btv710

[69] Maretty L , Jensen JM , Petersen B , Sibbesen JA , Liu S , Villesen P , et al. Sequencing and de novo assembly of 150 genomes from Denmark as a population reference. Nature 2017;548:87–91. 28746312 10.1038/nature23264

[70] Sibbesen JA , Maretty L , Krogh A . Accurate genotyping across variant classes and lengths using variant graphs. Nat Genet 2018;50:1054–9. 29915429 10.1038/s41588-018-0145-5

[71] Cai L , Yuan W , Zhang Z , He L , Chou KC . In‐depth comparison of somatic point mutation callers based on different tumor next‐generation sequencing depth data. Sci Rep 2016;6:36540. 27874022 10.1038/srep36540 PMC5118795

[72] Oesper L , Satas G , Raphael BJ . Quantifying tumor heterogeneity in whole‐genome and whole‐exome sequencing data. Bioinformatics 2014;30:3532–40. 25297070 10.1093/bioinformatics/btu651 PMC4253833

[73] Easton DF , Pharoah PD , Antoniou AC , Tischkowitz M , Tavtigian SV , Nathanson KL , et al. Gene‐panel sequencing and the prediction of breast‐cancer risk. N Engl J Med 2015;372:2243–57. 26014596 10.1056/NEJMsr1501341 PMC4610139

[74] Grindedal EM , Heramb C , Karsrud I , Ariansen SL , Maehle L , Undlien DE , et al. Current guidelines for BRCA testing of breast cancer patients are insufficient to detect all mutation carriers. BMC Cancer 2017;17:438. 28637432 10.1186/s12885-017-3422-2 PMC5480128

[75] Jonasson JG , Stefansson OA , Johannsson OT , Sigurdsson H , Agnarsson BA , Olafsdottir GH , et al. Oestrogen receptor status, treatment and breast cancer prognosis in Icelandic BRCA2 mutation carriers. Br J Cancer 2016;115:776–83. 27537391 10.1038/bjc.2016.249 PMC5046206

[76] Li X , You R , Wang X , Liu C , Xu Z , Zhou J , et al. Effectiveness of prophylactic Surgeries in BRCA1 or BRCA2 mutation carriers: a meta‐analysis and systematic review. Clin Cancer Res 2016;22:3971–81. 26979395 10.1158/1078-0432.CCR-15-1465

[77] Nilsson MP , Winter C , Kristoffersson U , Rehn M , Larsson C , Saal LH , et al. Efficacy versus effectiveness of clinical genetic testing criteria for BRCA1 and BRCA2 hereditary mutations in incident breast cancer. Fam Cancer 2017;16:187–93. 28120249 10.1007/s10689-016-9953-x PMC5357494

[78] Goodwin PJ , Phillips KA , West DW , Ennis M , Hopper JL , John EM , et al. Breast cancer prognosis in BRCA1 and BRCA2 mutation carriers: an International Prospective Breast Cancer Family Registry population‐based cohort study. J Clin Oncol 2012;30:19–26. 22147742 10.1200/JCO.2010.33.0068

[79] Hahnen E , Lederer B , Hauke J , Loibl S , Krober S , Schneeweiss A , et al. Germline mutation status, pathological complete response, and disease‐free survival in triple‐negative breast cancer: secondary analysis of the GeparSixto randomized clinical trial. JAMA Oncol 2017;3:1378–85. 28715532 10.1001/jamaoncol.2017.1007 PMC5710508

[80] Litton JK , Rugo HS , Ettl J , Hurvitz SA , Goncalves A , Lee KH , et al. Talazoparib in patients with advanced breast cancer and a germline BRCA mutation. N Engl J Med 2018;379:753–63. 30110579 10.1056/NEJMoa1802905 PMC10600918

[81] Robson M , Im SA , Senkus E , Xu B , Domchek SM , Masuda N , et al. Olaparib for metastatic breast cancer in patients with a germline BRCA mutation. N Engl J Med 2017;377:523–33. 28578601 10.1056/NEJMoa1706450

[82] Taylor A , Brady AF , Frayling IM , Hanson H , Tischkowitz M , Turnbull C , et al. Consensus for genes to be included on cancer panel tests offered by UK genetics services: guidelines of the UK Cancer Genetics Group. J Med Genet 2018;55:372–7. 29661970 10.1136/jmedgenet-2017-105188 PMC5992364

[83] Southey MC , Park DJ , Nguyen‐Dumont T , Campbell I , Thompson E , Trainer AH , et al. COMPLEXO: identifying the missing heritability of breast cancer via next generation collaboration. Breast Cancer Res 2013;15:402. 23809231 10.1186/bcr3434 PMC3706918

[84] Nielsen FC , van Overeem HT , Sørensen CS . Hereditary breast and ovarian cancer: new genes in confined pathways. Nat Rev Cancer 2016;16:599. 27515922 10.1038/nrc.2016.72

[85] Prakash R , Zhang Y , Feng W , Jasin M . Homologous recombination and human health: the roles of BRCA1, BRCA2, and associated proteins. Cold Spring Harb Perspect Biol 2015;7:a016600. 25833843 10.1101/cshperspect.a016600 PMC4382744

[86] Jiang Q , Greenberg RA . Deciphering the BRCA1 tumor suppressor network. The Journal of Biological Chemistry 2015;290:17724–32. 26048987 10.1074/jbc.R115.667931 PMC4505021

[87] Shakya R , Szabolcs M , McCarthy E , Ospina E , Basso K , Nandula S , et al. The basal‐like mammary carcinomas induced by Brca1 or Bard1 inactivation implicate the BRCA1/BARD1 heterodimer in tumor suppression. Proc Natl Acad Sci USA 2008;105:7040–5. 18443292 10.1073/pnas.0711032105 PMC2365565

[88] Stracker TH , Petrini JH . The MRE11 complex: starting from the ends. Nat Rev Mol Cell Biol 2011;12:90–103. 21252998 10.1038/nrm3047 PMC3905242

[89] Shiloh Y . ATM: expanding roles as a chief guardian of genome stability. Exp Cell Res 2014;329:154–61. 25218947 10.1016/j.yexcr.2014.09.002

[90] Bonadona V , Bonaiti B , Olschwang S , Grandjouan S , Huiart L , Longy M , et al. Cancer risks associated with germline mutations in MLH1, MSH2, and MSH6 genes in Lynch syndrome. JAMA 2011;305:2304–10. 21642682 10.1001/jama.2011.743

[91] Engel C , Loeffler M , Steinke V , Rahner N , Holinski‐Feder E , Dietmaier W , et al. Risks of less common cancers in proven mutation carriers with lynch syndrome. J Clin Oncol 2012;30:4409–15. 23091106 10.1200/JCO.2012.43.2278

[92] de Wind N , Dekker M , Berns A , Radman M , te Riele H . Inactivation of the mouse Msh2 gene results in mismatch repair deficiency, methylation tolerance, hyperrecombination, and predisposition to cancer. Cell 1995;82:321–30. 7628020 10.1016/0092-8674(95)90319-4

[93] Alemayehu A , Fridrichova I . The MRE11/RAD50/NBS1 complex destabilization in Lynch‐syndrome patients. Eur J Hum Genet 2007;15:922–9. 17534377 10.1038/sj.ejhg.5201858

[94] Shaltiel IA , Krenning L , Bruinsma W , Medema RH . The same, only different – DNA damage checkpoints and their reversal throughout the cell cycle. J Cell Sci 2015;128:607–20. 25609713 10.1242/jcs.163766

[95] Smith J , Tho LM , Xu N , Gillespie DA . The ATM‐Chk2 and ATR‐Chk1 pathways in DNA damage signaling and cancer. Adv Cancer Res 2010;108:73–112. 21034966 10.1016/B978-0-12-380888-2.00003-0

[96] Gonzalez KD , Noltner KA , Buzin CH , Gu D , Wen‐Fong CY , Nguyen VQ , et al. Beyond Li Fraumeni Syndrome: clinical characteristics of families with p53 germline mutations. J Clin Oncol 2009;27:1250–6. 19204208 10.1200/JCO.2008.16.6959

[97] McBride KA , Ballinger ML , Killick E , Kirk J , Tattersall MH , Eeles RA , et al. Li‐Fraumeni syndrome: cancer risk assessment and clinical management. Nat Rev Clin Oncol 2014;11:260–71. 24642672 10.1038/nrclinonc.2014.41

[98] Canman CE , Lim DS , Cimprich KA , Taya Y , Tamai K , Sakaguchi K , et al. Activation of the ATM kinase by ionizing radiation and phosphorylation of p53. Science 1998;281:1677–9. 9733515 10.1126/science.281.5383.1677

[99] Meijers‐Heijboer H , van den Ouweland A , Klijn J , Wasielewski M , de Snoo A , Oldenburg R , et al. Low‐penetrance susceptibility to breast cancer due to CHEK2(*)1100delC in noncarriers of BRCA1 or BRCA2 mutations. Nat Genet 2002;31:55–9. 11967536 10.1038/ng879

[100] Weischer M , Nordestgaard BG , Pharoah P , Bolla MK , Nevanlinna H , Van't Veer LJ , et al. CHEK2*1100delC heterozygosity in women with breast cancer associated with early death, breast cancer‐specific death, and increased risk of a second breast cancer. J Clin Oncol 2012;30:4308–16. 23109706 10.1200/JCO.2012.42.7336 PMC3515767

[101] Davies H , Glodzik D , Morganella S , Yates LR , Staaf J , Zou X , et al. HRDetect is a predictor of BRCA1 and BRCA2 deficiency based on mutational signatures. Nat Med 2017;23:517–25. 28288110 10.1038/nm.4292 PMC5833945

[102] Ledermann J , Harter P , Gourley C , Friedlander M , Vergote I , Rustin G , et al. Olaparib maintenance therapy in platinum‐sensitive relapsed ovarian cancer. N Engl J Med 2012;366:1382–92. 22452356 10.1056/NEJMoa1105535

[103] Mateo J , Carreira S , Sandhu S , Miranda S , Mossop H , Perez‐Lopez R , et al. DNA‐repair defects and olaparib in metastatic prostate cancer. N Engl J Med 2015;373:1697–708. 26510020 10.1056/NEJMoa1506859 PMC5228595

[104] Davies H , Morganella S , Purdie CA , Jang SJ , Borgen E , Russnes H , et al. Whole‐genome sequencing reveals breast cancers with mismatch repair deficiency. Can Res 2017;77:4755–62. 10.1158/0008-5472.CAN-17-1083 28904067

